# Disulfidptosis: The metabolic Achilles’ heel of SLC7A11-high tumors and why oncology has missed it

**DOI:** 10.1097/MS9.0000000000005117

**Published:** 2026-05-07

**Authors:** Mirza Muhammad Hadeed Khawar, Irum Naeem, Sifat Ullah Safi, Muneeb Khawar

**Affiliations:** a Department of Medicine, Services Institute of Medical Sciences, Lahore, Pakistan; b Department of Medicine, King Edward Medical University, Lahore, Pakistan; c Department of Medicine, Kabul Medical University, Kabul, Afghanistan

**Keywords:** actin cytoskeleton, disulfidptosis, glucose deprivation, NADPH, precision oncology, SLC7A11

## Abstract

SLC7A11 overexpression is one of the most consistent features of treatment-resistant solid tumors, yet its metabolic liabilities remain therapeutically unexploited. Tumors that upregulate SLC7A11 import large quantities of cystine to fuel glutathione synthesis and resist ferroptosis, a strategy that renders them addicted to glucose-derived NADPH for disulfide reduction. Under glucose limitation, NADPH depletion causes toxic cystine accumulation, aberrant disulfide bonding of actin cytoskeletal proteins, F-actin collapse, and a distinct form of regulated cell death termed disulfidptosis. First characterized in 2023, disulfidptosis operates independently of caspases, lipid peroxidation, and reactive oxygen species, and cannot be rescued by inhibitors of apoptosis, ferroptosis, or necroptosis. Recent evidence shows that this pathway extends beyond cancer cells: intratumoral CD8+ T cells undergo SLC7A11-dependent disulfidptosis, contributing to T cell exhaustion and immune evasion. Glucose transporter inhibitors such as BAY-876 selectively trigger disulfidptosis in SLC7A11-high patient-derived xenografts while sparing SLC7A11-low tumors. Despite this, no clinical oncology framework currently stratifies patients by SLC7A11-dependent metabolic vulnerability, and no disulfidptosis-targeting agent has entered clinical trials. Challenges include the absence of validated *in vivo* biomarkers for disulfide stress, the dual role of SLC7A11 in both tumor survival and immune cell death, and an incomplete understanding of how tumor microenvironmental glucose availability modulates disulfidptosis thresholds. This Letter to the Editor argues that disulfidptosis is an actionable metabolic vulnerability that precision oncology has overlooked and proposes a framework for its clinical translation.

*Dear Editor*,

Resistance to cell death is a defining hallmark of cancer, and for the past decade, ferroptosis has dominated the regulated cell death conversation in oncology. Tumors frequently circumvent ferroptosis by overexpressing SLC7A11 (xCT), the cystine/glutamate antiporter that sustains intracellular glutathione pools[1]. This observation has driven a sustained search for ferroptosis-inducing agents. What the field has largely ignored, however, is what happens on the other side of the equation: SLC7A11-high tumors import massive quantities of cystine, and reducing that cystine to cysteine costs NADPH. When glucose is scarce, and the pentose phosphate pathway falters, NADPH runs out, cystine accumulates in its oxidized disulfide form, and the cell dies – not by ferroptosis, but by a mechanistically distinct pathway that Liu *et al* termed disulfidptosis in 2023[1].

The molecular logic is specific. Unreduced cystine forms aberrant disulfide bonds with actin cytoskeletal proteins, including members of the Rac1-WAVE regulatory complex that drives lamellipodia formation. The branched actin network collapses, F-actin contracts and detaches from the plasma membrane, and the cell undergoes rapid death[1]. Inhibitors of apoptosis, necroptosis, and ferroptosis do nothing to stop this process. CRISPR screens confirmed that knocking out any WAVE regulatory complex subunit partially rescues cells, while glucose transporter (GLUT) inhibition with BAY-876 potently triggers disulfidptosis in SLC7A11-high but not SLC7A11-low patient-derived xenograft models[1]. The vulnerability is metabolically conditional: it requires high SLC7A11, low glucose, and insufficient NADPH, conditions that frequently coexist in the hypoxic, nutrient-deprived tumor core.

Two recent advances broaden this framework. Wan *et al* showed that disulfidptosis is not confined to cancer cells[2]. Tumor-infiltrating CD8+ T cells also take up cystine via SLC7A11, and under the glucose-depleted conditions of the tumor microenvironment, lactate dehydrogenase B restricts glucose-6-phosphate dehydrogenase activity in exhausted T cells, depleting NADPH and triggering disulfidptosis[2]. This reframes the problem entirely: SLC7A11 inhibition could simultaneously induce ferroptosis in cancer cells and protect T cells from disulfide stress-mediated exhaustion, but the therapeutic window is narrow and context dependent. Separately, Gan consolidated evidence in Nature Reviews Molecular Cell Biology that disulfidptosis can also be triggered under oxidative conditions (e.g., H_2_O_2_ exposure) in SLC7A11-high cells independent of glucose starvation, broadening the biological contexts in which this pathway operates[3].

These two findings sit in contrast. One reveals disulfidptosis as a route to tumor cell killing. The other identifies it as a mechanism of immune sabotage. They are not contradictory; they describe the same metabolic fragility manifesting in different cellular compartments. Any therapy designed around disulfidptosis must therefore account for this immunological collateral.

Several obstacles stand between these mechanistic insights and clinical utility. There is no validated biomarker for disulfide stress in human tumor tissue; malondialdehyde and 4-hydroxynonenal reflect lipid peroxidation (ferroptosis), not disulfide bond accumulation. SLC7A11 expression alone is an imperfect surrogate because it fails to capture microenvironmental glucose availability or NADPH flux[4]. Animal models of disulfidptosis have relied on GLUT inhibitors that are not yet clinically available, and the interplay between disulfidptosis and ferroptosis within the same tumor remains poorly mapped[4]. The thioredoxin reductase (TrxR1) axis adds a further complication: Tang *et al* showed that TrxR1 inhibition sensitizes glucose-starved glioblastoma cells to disulfidptosis, suggesting that the thioredoxin system provides a parallel disulfide-buffering capacity that tumors may exploit for resistance[5].

Precision oncology needs a metabolic vulnerability classification that goes beyond mutational profiling. To our knowledge, no existing clinical oncology framework stratifies patients by SLC7A11-dependent metabolic vulnerability, a gap recently underscored by comprehensive analyses of metabolic biomarker integration in solid tumor trials[6]. We propose stratifying tumors by SLC7A11 expression combined with intratumoral glucose and NADPH metabolism, measurable via 18F-FDG-PET heterogeneity and pentose phosphate pathway gene signatures, to identify disulfidptosis-vulnerable populations. The feasibility of detecting aberrant disulfide bonding in clinical specimens is supported by advances in redox proteomics and plasma metabolomics: iodoacetamide-based cysteine reactivity profiling can quantify disulfide-modified actin peptides in tumor biopsies, and untargeted metabolomic platforms have detected cystine accumulation signatures in patient plasma, providing a precedent for non-invasive disulfide stress monitoring[7]. A rational pilot trial design would pair GLUT or TrxR1 inhibitors with immune checkpoint blockade in SLC7A11-high solid tumors, monitoring both tumor regression and CD8+ T cell functional status as co-primary endpoints. An important caveat concerns the off-target toxicity of GLUT inhibition: glucose is the primary fuel for the brain, erythrocytes, and renal medulla, and systemic GLUT1 blockade with agents such as BAY-876 risks neuroglycopenia and hematological toxicity at supra-therapeutic doses[8]. Consequently, intermittent dosing schedules, tumor-targeted GLUT1 nanoformulations, or preferential use of TrxR1 inhibitors in brain-adjacent tumors should be considered in early-phase trial design to preserve a viable therapeutic window. Until oncology integrates disulfide stress biology into its therapeutic framework, the metabolic Achilles’ heel that SLC7A11 overexpression creates will remain unharnessed.

## Data Availability

Not applicable.

## References

[1] Liu X Nie L Zhang Y. Actin cytoskeleton vulnerability to disulfide stress mediates disulfidptosis. Nat Cell Biol 2023;25:404–14. 36747082 10.1038/s41556-023-01091-2 PMC10027392

[2] Wan J Shi JH Shi M. Lactate dehydrogenase B facilitates disulfidptosis and exhaustion of tumour-infiltrating CD8+ T cells. Nat Cell Biol 2025;27:972–82. 40461882 10.1038/s41556-025-01673-2

[3] Gan B. Redox-driven cell death by disulfidptosis and its therapeutic potential. Nat Rev Mol Cell Biol 2025;26:727–29. 40847224 10.1038/s41580-025-00888-3

[4] Liu X Zhuang L Gan B. Disulfidptosis: disulfide stress–induced cell death. Trends Cell Biol 2024;34:327–37. 37574347 10.1016/j.tcb.2023.07.009

[5] Tang M Dirks K Kim SY. Inhibition of thioredoxin reductase 1 sensitizes glucose-starved glioblastoma cells to disulfidptosis. Cell Death Differ 2025;32:598–612. 39715824 10.1038/s41418-024-01440-0 PMC11982235

[6] Reinfeld BI Madden MZ Wolf MM. Cell-programmed nutrient partitioning in the tumour microenvironment. Nature 2021;593:282–88. 33828302 10.1038/s41586-021-03442-1 PMC8122068

[7] Bak DW Weerapana E. Cysteine-mediated redox signalling in the mitochondria. Mol Biosyst 2015;11:678–97. 25519845 10.1039/c4mb00571f

[8] Ancey PB Contat C Meylan E. Glucose transporters in cancer–from tumor cells to the tumor microenvironment. FEBS J 2018;285:2926–43. 29893496 10.1111/febs.14577

